# NDVI and Beyond: Vegetation Indices as Features for Crop Recognition and Segmentation in Hyperspectral Data †

**DOI:** 10.3390/s25123817

**Published:** 2025-06-18

**Authors:** Andreea Nițu, Corneliu Florea, Mihai Ivanovici, Andrei Racoviteanu

**Affiliations:** 1 AI4AGRI, Romanian Excellence Center on AI for Agriculture, Transilvania University of Brasov, 500024 Brasov, Romania; andreea.nitu3112@upb.ro (A.N.); mihai.ivanovici@unitbv.ro (M.I.); andrei.racoviteanu@upb.ro (A.R.); 2 Image Processing and Analysis Laboratory, National University of Science and Technology Politehnica Bucharest, Splaiul Independentei 313, 060042 Bucharest, Romania

**Keywords:** vegetation indices, NDVI, remote sensing, hyperspectral imaging, classification, similarity metrics

## Abstract

Vegetation indices have long been central to vegetation monitoring through remote sensing. The most popular one is the Normalized Difference Vegetation Index (NDVI), yet many vegetation indices (VIs) exist. In this paper, we investigate their distinctiveness and discriminative power in the context of applications for agriculture based on hyperspectral data. More precisely, this paper merges two complementary perspectives: an unsupervised analysis with PRISMA satellite imagery to explore whether these indices are truly distinct in practice and a supervised classification over UAV hyperspectral data. We assess their discriminative power, statistical correlations, and perceptual similarities. Our findings suggest that while many VIs have a certain correlation with the NDVI, meaningful differences emerge depending on landscape and application context, thus supporting their effectiveness as discriminative features usable in remote crop segmentation and recognition applications.

## 1. Introduction

While the modern human has become more and more sophisticated, with more elevated needs, the need for food remains fundamental. Due to increasing global food demand, climate variability, and the need for sustainable resource management, it has become imperative to deploy smart tools that optimize crop yield and reduce ecological footprints. Among the emerging technologies shaping modern precision agriculture, remote sensing (RS) has proven to be one of the most transformative [1]. RS techniques enable farmers, scientists, and policymakers to remotely monitor, evaluate, and predict vegetation health and land-use changes across vast geographical regions in real time [2].

Vegetation indices (VIs) constitute a foundational element in remote sensing-based agricultural analysis, providing rapid, scalable, and interpretable insights into vegetation health, biomass, and canopy dynamics [3]. Over the past few decades, a plethora of indices have been developed—each intended to capture particular spectral characteristics of vegetation reflective properties under specific environmental or phenological conditions. We kindly refer the reader to the study of Ivanovici et al. [4] for a more thorough and recent presentation, and to the studies of Tucker [5] or Curran [6] for some initial views. From the commonly used Normalized Difference Vegetation Index (NDVI) [7] to more specific ones like the Atmospherically Resistant Vegetation Index (ARVI) [8] or the Visible Atmospherically Resistant Index (VARI) [9], there are many different vegetation indices available. Although often included in such studies, it must be highlighted that Leaf Area Index (LAI) [10] is not a vegetation index but a canopy biophysical parameter, yet due to its popularity we will also include it. This section offers a comprehensive overview of key prior contributions regarding the comparative evaluation of VIs, their discriminative and correlational behavior, and the methodologies employed in such investigations, including both supervised and unsupervised paradigms.

The research presented in this paper combines supervised learning with unsupervised analytical techniques to evaluate how vegetation indices perform when used in isolation and in comparison. We leverage two rich data sources: hyperspectral satellite imagery from the PRISMA mission and a high-resolution UAV-based hyperspectral dataset known as UAV-HSI [11]. The PRISMA satellite provides hyperspectral imagery across 240 contiguous spectral bands ranging from 400 to 2500 nm, with a spectral resolution of approximately 10 nm. Its spatial resolution is 30 m for hyperspectral data. The UAV-based hyperspectral imaging system captures data in the visible to near-infrared range (typically 400–1000 nm) using 40 to 100 spectral bands, depending on the sensor configuration. It offers ultra-high spatial resolution, often below 10 cm per pixel, allowing detailed analysis of small-scale features. By examining both pixel-level classification tasks and image-wide statistical behavior, we capture a comprehensive view of how VIs behave across multiple spatial scales.

The PRISMA imagery allows us to assess VI behavior in diverse ecosystems, including forested regions, alpine pastures, cultivated fields, and urbanized areas. Using unsupervised tools—Pearson and Spearman correlation coefficients, Euclidean distance metrics, and image similarity scores (SSIMs)—we quantify the relationships and perceptual consistency between NDVI and eight popular VIs, DVI, SAVI, VARI, GNDVI, ARVI, EVI, GEMI, and GARI, to which we add a ninth, LAI.

We selected these VIs for our experiments due to their diverse sensitivity to key biophysical and environmental conditions relevant to vegetation monitoring. These indices cover a broad range of spectral combinations and mathematical formulations, allowing us to capture variations in canopy structure (e.g., LAI, GEMI), soil background effects (e.g., SAVI), atmospheric resistance (e.g., ARVI, EVI), and chlorophyll content (e.g., GNDVI, GARI). Their widespread adoption and diverse characteristics make them a robust and balanced choice for evaluating vegetation across different land cover types and sensor platforms.

Meanwhile, the UAV-HSI dataset enables us to simulate practical, precision agriculture scenarios. Collected using a hyperspectral sensor mounted on a UAV, the dataset contains 200 spectral bands and pixel-wise crop annotations across 30 vegetation classes. Through supervised classification using Random Forest (RF), Multilayer Perceptron (MLP), Support Vector Machine (SVM) and Gradient Boosting Machine (GBM) models, we explore how well the enumerated VIs alone can support crop identification and how their importance ranks in feature selection.

A major strength of this dual-perspective methodology is that it captures both functional and contextual differences. Unsupervised analysis helps identify statistical redundancy or complementary behavior among indices, while supervised classification highlights how useful a VI is as a discriminative feature descriptor. Together, these perspectives contribute to a holistic understanding of how VIs operate in real-world settings. In this paper we make use of machine learning and data analytic tools to delve into the question of whether ViS are good descriptors.

In this paper, we present an in-depth comparison of 10 major vegetation indices in terms of classification power and statistical similarity. We analyze how the performance of each index is influenced by terrain type and environmental variability. The term “terrain type” in this study refers to four main natural relief categories: pasture, forest, agricultural land, and urban areas, as defined by the PRISMA land cover annotations. Environmental variability, in this context, captures the temporal differences in vegetation condition, primarily driven by phenological stages such as emergence and growth. This paper continues and extends two of our previous conference works [12,13]. This work develops by considering a broader list of VIs and a more detailed evaluation, which is as follows: on the unsupervised part, we extend [12] by considering more data (i.e., more robust and inclusive annotation) and more VIs; on the supervised part, we extend [13] by considering more classifiers and additional feature ranking algorithms, leading to an overall more thorough discussion.

### 1.1. Prior Work

Phenological data analysis and discrimination has long been an interesting theme in the community [14,15,16] and many directions have been approached.

#### 1.1.1. The Emergence of Vegetation Indices

Vegetation indices (VIs) have emerged as one of the most accessible and interpretable outputs of remote sensing (RS) data [3]. They are mathematical transformations of spectral reflectance values—typically in the visible (VIS) and near-infrared (NIR) spectral bands—designed to highlight vegetation characteristics such as chlorophyll concentration, canopy structure, and water content. The development of VIs stems from the need to distill high-dimensional spectral information, especially in hyperspectral imagery, into biologically relevant and computationally tractable descriptors [17].

Among the earliest and most widely used indices is the Normalized Difference Vegetation Index (NDVI), which exploits the difference in reflectance between the red and NIR bands to estimate vegetation vigor [17,18]. NDVI is prized for its simplicity and general applicability but exhibits limitations in conditions involving dense vegetation, atmospheric interference, or bright soil backgrounds [18].

To address such limitations, various alternative indices have been developed. The Enhanced Vegetation Index (EVI) was designed to reduce sensitivity to soil and atmospheric noise, making it more effective in high-biomass areas. The Green NDVI (GNDVI) replaces the red band with the green band to improve sensitivity to chlorophyll content. The Atmospherically Resistant Vegetation Index (ARVI) incorporates a blue band correction to reduce atmospheric scattering, proving more robust under hazy or polluted conditions [19]. Meanwhile, the Soil-Adjusted Vegetation Index (SAVI) includes a soil brightness correction factor, enhancing performance in arid and semi-arid regions [20].

Given their expert-driven formulation, VIs have been widely adopted in agricultural remote sensing, enabling tasks ranging from yield prediction, irrigation scheduling, and crop classification to change detection and phenological monitoring [3,21]. However, the very proliferation of VIs also introduced a challenge: are these indices truly offering complementary information, or are many of them effectively redundant?

#### 1.1.2. Quantitative Comparisons and Discriminative Evaluation

Multiple studies have aimed to evaluate the effectiveness of vegetation indices (VIs), often using supervised learning or regression-based frameworks. For instance, Payero et al. [22] examined 11 VIs and assessed their correlation with plant height in crops such as alfalfa and grass. They observed moderate to strong correlations for all tested indices, with TVI, NDVI, and IPVI standing out, highlighting both the robustness and phenological sensitivity of VIs. Similarly, Cao et al. [23] investigated index saturation under dense canopy conditions, showing that the NDVI saturates earlier than alternatives like SAVI and VIUPD, especially in mountainous terrains. This underscored the need for indices with an extended linear response range. Vina et al. [24] conducted a biophysically grounded comparison of indices for estimating green biomass, while Towers et al. [25] evaluated VI performance in vineyards, finding NDVI optimal for linear models but SAVI2 more effective during specific phenological stages under non-linear models. These studies collectively emphasize that VI performance is highly context-sensitive—dependent on crop type, landscape, and environmental conditions—and often difficult to generalize across broader settings.

Each index brings an unique perspective to vegetation monitoring and reflects expert knowledge developed for specific spectral and biophysical scenarios. In this sense, VIs are “expert features” rooted in the expert systems paradigm [26]. Their performance, however, may vary depending on temporal resolution, sensor configuration, or terrain complexity [3]. This variability poses a practical challenge: how should one select the most appropriate index for a specific application? While NDVI remains a go-to choice, alternatives such as VARI or LAI may be more suitable in particular environments or use cases. For example, in yield forecasting, the choice of VI can directly impact model accuracy and inform resource allocation [27]. In early drought detection or disease monitoring, the sensitivity and specificity of an index can determine whether interventions are timely or missed altogether [28].

Despite the proliferation of VIs, many comparative studies remain narrow in scope or limited in methodology. Some rely only on visual comparisons or basic correlation statistics [29], while others evaluate just a few indices in isolation [30,31]. To our best knowledge, no study has yet proposed a multi-angle approach that combines supervised and unsupervised analysis across different platforms, spatial resolutions, and land cover types. This paper aims to fill that gap.

#### 1.1.3. Vegetation Indices in Supervised Machine Learning Contexts

Recent advances in remote sensing and machine learning have encouraged the re-evaluation of VIs from a supervised classification perspective. Simsek [31] optimized spectral and textural indices for crop classification by considering spatial heterogeneity; using methods like Pearson’s correlation coefficient and the Recursive Feature Addition Algorithm (RFAA), this study reduced redundancy among indices and improved classification efficiency. Najar et al. [32] trained a deep neural network on multispectral satellite data and analyzed feature importance, identifying the most influential spectral bands; then they modified existing VIs to incorporate these bands, leading to improved classification performance. Asgari et al. [30] evaluated the performance of three machine learning classifiers—Random Forest (RF), Gradient Boosting (GB), and K-Nearest Neighbors (KNN)—in mapping crop types using various VIs derived from Sentinel-2 imagery.

### 1.2. Contextual Sensitivity and Terrain-Dependent Behavior

A recurring theme across the prior literature is the sensitivity of VI performance to contextual factors [33]. These include the following:

- Crop type and phenological stage: Certain indices may respond more strongly during early growth stages or under specific stress conditions. For instance, Rose et al. demonstrated the necessity for crop-specific calibrations when estimating parameters like green area index and nitrogen uptake [34].
- Soil background and moisture: Indices like SAVI were developed specifically to mitigate soil brightness influences, as highlighted by Huete [35].
- Geographic and topographic variation: Topographic factors such as slope and altitude can affect reflectance characteristics, influencing index reliability. Zhu et al. evaluated these effects on commonly used VIs [36].
- Sensor type and calibration: Differences in spectral resolution and acquisition geometry between UAVs and satellites can lead to significant variations in index values. Bukowiecki et al. emphasized the importance of sensor calibration in such contexts [34].

These factors collectively suggest that while VIs are rooted in physical models, their real-world performance cannot be decoupled from observational and environmental constraints.

### 1.3. Remaining Challenges and Research Gaps

Despite extensive prior work, several open questions remain in the comparative evaluation of VIs:

1. Lack of unified benchmarks: Studies often use different datasets, scales, and evaluation criteria, making it difficult to aggregate or generalize findings.
2. Over-reliance on linear metrics: Many prior analyses focus solely on Pearson correlation, overlooking non-linear dependencies or structural variation in VI data.
3. Insufficient integration of supervised and unsupervised views: Few studies combine discriminative evaluation with redundancy assessment, though such integration may yield richer insights.
4. Interpretability versus accuracy trade-offs: While VIs are interpretable and computationally light, they often underperform against raw spectral data in black-box models like deep neural networks. The trade-offs between explainability and performance remain under-explored.

Our paper tries to alleviate some of these issues.

## 2. Materials and Methods

This section describes the methodological framework employed to assess and compare the behavior of VIs from both supervised and unsupervised perspectives. Our goal is two-fold: (i) First we examine the statistical and perceptual similarity between popular indices using an unsupervised, correlation-based approach. The aim is to determine how much correlation and thus redundancy exists between indices. (ii) Secondly, we evaluate the discriminative power of various VIs using a supervised classification pipeline. To this end, we employ two distinct high-resolution hyperspectral datasets—UAV-based and satellite-based (PRISMA)—each offering complementary insights into the performance of VIs under different acquisition conditions, spatial resolutions, and land cover scenarios.

### 2.1. Overview of Approach

The overall methodology comprises the following key steps:

1. Computation of a representative set of VIs using established spectral formulas.
2. Assessment of inter-index similarity using statistical and perceptual metrics in an unsupervised framework, conditioned on land cover type.
3. Evaluation of their effectiveness as features in a supervised learning setting (multi-class and binary segmentation of crop types).
4. Comparative analysis of index behavior across multiple scenarios—unsupervised analysis (e.g., statistical correlations, SSIM) and supervised classification (e.g., Random Forest, SVM), sensors, and semantic categories.

Each of these steps is detailed in the subsections below.

### 2.2. Vegetation Indices Considered

We selected a subset of ten VIs that are commonly employed in precision agriculture and environmental monitoring. These indices were chosen to provide spectral diversity, robustness to atmospheric and soil effects, and sensitivity to various plant physiological traits. They include the following:

- The **Normalized Difference Vegetation Index (NDVI)** is a widely used remote sensing index that measures live green vegetation (being sensitive to chlorophyll) and is computed as follows:
  (1) $$\mathrm{NDVI}=\frac{\mathrm{NIR}-\mathrm{Red}}{\mathrm{NIR}+\mathrm{Red}}$$
  NDVI values range from −1 to +1 and their interpretation is as follows:
  – Values close to +1 (e.g., 0.6–0.9) indicate dense, healthy vegetation.
  – Values around 0 to 0.2 typically correspond to bare soil, rocks, or dead vegetation.
  – Negative values usually indicate water, snow, or clouds.
- **Soil-Adjusted Vegetation Index (SAVI)** [35]
  (2) $$\mathrm{SAVI}=\frac{\left(\mathrm{NIR}-\mathrm{Red})\times (1+L\right)}{\left(\mathrm{NIR}+\mathrm{Red}+L\right)}$$
  where L=0.5. The SAVI helps isolate the vegetation signal from background soil, improving vegetation monitoring in heterogeneous landscapes. With respect to the *L* parameter, L=0 makes the SAVI identical to the NDVI, while L=1 is recommended in very-low-vegetation areas. As we aim to form discriminative VIs, *L* was set to an intermediary value.
- The **Green Normalized Difference Vegetation Index (GNDVI)** [9] is defined as follows:
  (3) $$\mathrm{GNDVI}=\frac{\mathrm{NIR}-\mathrm{Green}}{\mathrm{NIR}+\mathrm{Green}}$$
  The GNDVI was introduced to complement the NDVI as the latter uses the red band, which is absorbed strongly by chlorophyll; in contrast, the GNDVI uses the green band, which is reflected more by healthy vegetation. Thus, the GNDVI tends to be more sensitive to chlorophyll content and less sensitive to soil background than the NDVI.
- The **Difference Vegetation Index (DVI)** [37] was among the first VIs used in remote sensing, especially in the early days of satellite imagery like Landsat MSS in the 1970s. It is computed as follows:
  (4) DVI=NIR−Red
  Unlike the NDVI, the DVI does not normalize the values, so it is sensitive to overall brightness, illumination conditions, and atmospheric effects. The DVI is directly proportional to the amount of vegetation: higher values typically indicate denser or healthier vegetation. It is best used when conditions across the dataset are consistent, since a lack of normalization can lead to skewed results under variable lighting or soil conditions.
- The **Enhanced Vegetation Index (EVI)** [38] is designed to be more sensitive than the NDVI in dense vegetation (the NDVI tends to saturate), correct for atmospheric and soil background effects, and be more useful for high-biomass regions (e.g., forests). It is computed as follows:
  (5) $$\mathrm{EVI}=2.5\times \frac{\mathrm{NIR}-\mathrm{Red}}{\mathrm{NIR}+6\times \mathrm{Red}-7.5\times \mathrm{Blue}+1}$$
- The **Leaf Area Index (LAI)** [39] is a fundamental biophysical parameter used to describe vegetation. It quantifies the total leaf area per unit of ground surface area. However, in more recent works, this has been estimated via the Enhanced Vegetation Index (EVI), as it has been shown that they are empirically correlated [38,40]:
  (6) LAI=3.618×EVI−0.118
  We emphasize that the LAI is not a vegetation index, but a canopy biophysical parameter. From a biophysical point of view it should not be included; however, there are many prior works focusing on crop data analysis [3,25] that contrasted the LAI with other VIs. It is also very popular and, thus, our study includes it.
- The **Visible Atmospherically Resistant Index (VARI)** [41] is a VI designed to estimate vegetation greenness using only visible light bands (red, green, and blue). It is particularly useful when near-infrared (NIR) data is not available and is computed as follows:
  (7) $$\mathrm{VARI}=\frac{\mathrm{Green}-\mathrm{Red}}{\mathrm{Green}+\mathrm{Red}-\mathrm{Blue}}$$
  The VARI was designed to minimize atmospheric effects, especially those caused by scattering in the blue region. It correlates well with vegetation fraction and canopy greenness under certain conditions and it works best in open-canopy or moderate vegetation environments.
- The **Atmospherically Resistant Vegetation Index (ARVI)** [8] is a VI developed to reduce the influence of atmospheric effects, especially aerosols and atmospheric scattering, which commonly distort satellite reflectance in the visible bands. It is computed as follows:
  (8) $$\mathrm{ARVI}=\frac{\mathrm{NIR}-(\mathrm{Red}-\gamma (\mathrm{Blue}-\mathrm{Red}))}{\mathrm{NIR}+(\mathrm{Red}-\gamma (\mathrm{Blue}-\mathrm{Red}))}$$
  where γ=1.7. The term (Red−γ(Blue−Red)) replaces the red band used in the NDVI, correcting for atmospheric scattering (especially in the red band) using the blue band. Thus, the ARVI was designed to be more robust than the NDVI under hazy or smoky conditions.
- **Global Environmental Monitoring Index (GEMI)** [42] works in the same direction as ARVI and VARI by being a VI developed to improve the accuracy of vegetation monitoring by reducing the influence of atmospheric effects and soil background—issues that often affect indices like the NDVI and DVI. First, define
  (9) $$\eta =\frac{2({\mathrm{NIR}}^{2}-{\mathrm{Red}}^{2})+1.5\times \mathrm{NIR}+0.5\times \mathrm{Red}}{\mathrm{NIR}+\mathrm{Red}+0.5}$$
  Then:
  (10) $$\mathrm{GEMI}=\eta \left(1-0.25\times \eta \right)-\frac{\mathrm{Red}-0.125}{1-\mathrm{Red}}$$
  Like the NDVI, the GEMI is also sensitive to vegetation cover, but it performs better under low-vegetation conditions and variable atmospheric conditions. It is worth mentioning that the GEMI does not require the blue band or other atmospheric correction bands.
- The **Green Atmospherically Resistant Index (GARI)** [41] is computed as follows:
  (11) $$\mathrm{GARI}=\frac{\mathrm{NIR}-(\mathrm{Green}-\gamma (\mathrm{Blue}-\mathrm{Red}))}{\mathrm{NIR}+(\mathrm{Green}-\gamma (\mathrm{Blue}-\mathrm{Red}))}$$
  where γ=1.7. The GARI is a VI designed to monitor vegetation while being resistant to atmospheric effects, similar to the ARVI, but it uses the green band instead of the red. This makes it more sensitive to chlorophyll content and early signs of plant stress.

Each index was computed using its standard formulation based on reflectance values in the red, green, blue, and NIR bands. When computing indices from hyperspectral imagery, band selection was carefully aligned with the expected spectral ranges to ensure the fidelity of derived values. For the HSI-UAV dataset, the spectral bands are approximately as follows: red at 665 nm, near-infrared (NIR) at 789 nm, shortwave infrared (SWIR) at 1024 nm, blue at 424 nm, and green at 535 nm. In comparison, the PRISMA dataset bands are red at 653 nm, NIR at 779 nm, blue at 463 nm, and green at 537 nm. These wavelength values have been rounded to make the information simpler and more readable.

When the VIs are computed from multispectral (e.g., Sentinel-2) data, the spectral bands are quite large (tens or even hundreds of nm). Hyperspectral data offer higher spectral resolution and specificity. In our work, all formulas use the same spectral bands, and thus, although not obvious, there is the question whether VIs offer redundant descriptions of represented data. In certain cases, as is the linear relation between the EVI and LAI, redundancy is obvious, but in many cases there are no clear answers. To address this problem, we contribute with further investigations.

Another observation is that some of the VIs have been introduced to alleviate some issues faced by the NDVI in certain conditions. Based on this, we rely on the NDVI as the baseline while evaluating statistical dependency between VIs.

### 2.3. Datasets and Acquisition Platforms

In this paper we have utilized two datasets both acquired by hyperspectral sensors. The first one is a roughly annotated satellite (PRSIMA) set over the Brasov area, Romania. The second is the UAV-HSI dataset that has fine-grained annotations with respect to crops.

#### 2.3.1. PRISMA Dataset

**Figure 1:**
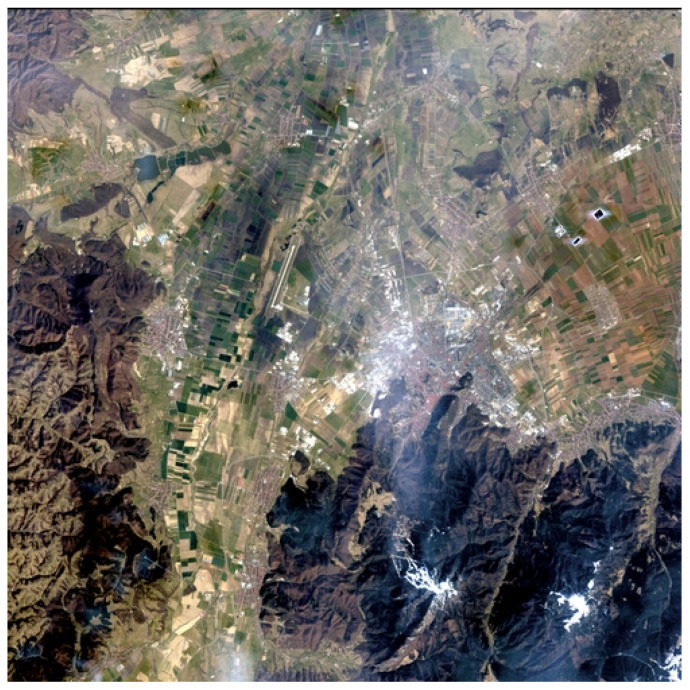
PRISMA hyperspectral image visualization on the date of 23 March 2024.

We utilized a hyperspectral scene acquired at four distinct time points (23rd March, 14th May, 18th June, and 30th June) by the PRISMA satellite over the Brașov city area in Romania. Pixels from all calendar dates were used jointly in the subsequent analysis. For visualization purposes, an RGB image captured on 23 March 2024 was rendered using the solution [43] and is shown in Figure 1. This solution presents a neural network-based method for visualizing multisource remote sensing data by mapping spectral inputs to RGB. A fully connected neural network was trained using interpolated and standardized data, with two preprocessing strategies—global and per-image normalization. Trained with Huber loss, the model produced consistent RGB outputs and generalized well to unseen datasets. The PRISMA sensor captures 187 spectral bands in the 400–2500 nm range, with a spatial resolution of 30 m.

The data was collected from the Potato Institute’s land in Brașov, located in the Bârsa premontane plain—a large depression in central Romania encircled by mountains. Known as part of Romania’s “Potato Country,” the Bârsa region spans 2406 km^2^, with altitudes ranging from 550 m (Bod) to 722 m (Zărnești), and lies between 45°27′–46°00′ N latitude and 26°10′–26°13′ E longitude.

This dataset was used to evaluate inter-index correlations in an unsupervised manner across semantically distinct regions—namely agricultural land, forest, alpine pasture, and urban areas. The separation of these classes is also based on the observations of the motivation and nature of VIs; namely, they have been designed to behave differently given different types of vegetation or terrain.

#### 2.3.2. UAV-HSI Dataset

**Figure 2:**
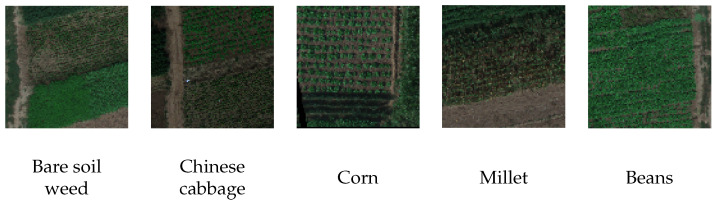
Examples of UAV-HSI hyperspectral images, rendered as RGB color components using a neural network-based method for visualizing multisource remote sensing data by mapping spectral inputs to RGB [43].

**Figure 3:**
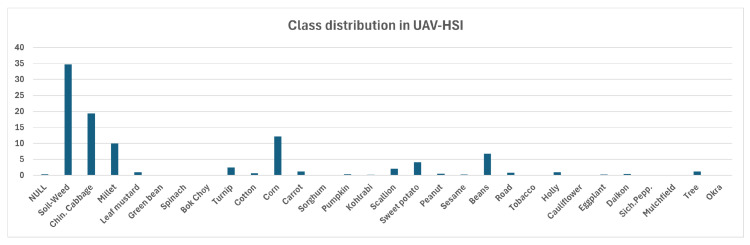
Class (pixel) distribution in the UAV-HSI dataset. *X* axis—crop type. *Y* axis—percentage of pixels with the respective crop.

The UAV-HSI dataset [11] provides high-resolution hyperspectral imagery acquired via a multirotor UAV equipped with a Pika L sensor, covering a spectral range of 385–1034 nm across 200 bands. This dataset is particularly suitable for evaluating supervised classification pipelines, as it contains extensive ground truth segmentation masks. In Figure 2, one may observe examples of images having the dominant culture marked. Following the methodology outlined in the introductory paper [11], the dataset was divided into training and testing subsets: 346 hyper-images were in the training and 87 in the testing set. Each image had a resolution of 96×96 pixels. The spatial resolution was sub-meter, and the dataset included pixel-level annotations for 30 agricultural crop classes, which are unevenly distributed as shown in Figure 3. The importance of the spectral bands is detailed in both the introductory paper and the camera manual, which also includes formulas for calculating VIs given the specific bands.

### 2.4. Unsupervised Evaluation: Inter-Index Similarity Analysis

An unsupervised evaluation was carried out in conjunction with PRISMA images. The purpose was to determine if VIs store non-redundant data that has enough discriminative power to describe objects in hyperspectral imaging and further enable separation between different types of vegetation.

#### 2.4.1. Semantic Zoning

**Figure 4:**
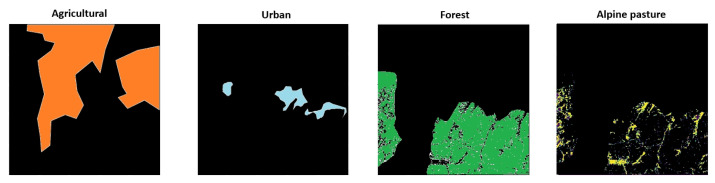
The masks over the PRISMA image that have been used for zone segmentation.

The PRISMA images over the Brașov city region were manually segmented into four semantic zones—agricultural crop, forest, alpine pasture, and urban—based on visual inspection and land cover characteristics. For each zone, VIs were computed and compared to the NDVI, which served as the reference. The image as well as the masks for each corresponding terrain can be seen in Figure 1 and Figure 4. Gathering data from all four images resulted in the following amount of data: agricultural terrain: 1,250,315 pixels, alpine pasture: 130,604 pixels, forest: 1,006,349 pixels, and urban landscape: 124,066 pixels.

Additionally, manual segmentation was performed during the data preprocessing stage to prohibit regions that were obscured by cloud coverage. After dividing the data into zones, the individual pixels were processed, and VIs were calculated using the original 187 raw bands. To prevent the dataset from containing invalid values such as “nan” or “inf, a correction value of 0.0001 was added to both the numerator and the denominator. To address corrupted values, those falling outside the expected range for each respective index, a clipping operation was applied.

**Figure 5:**
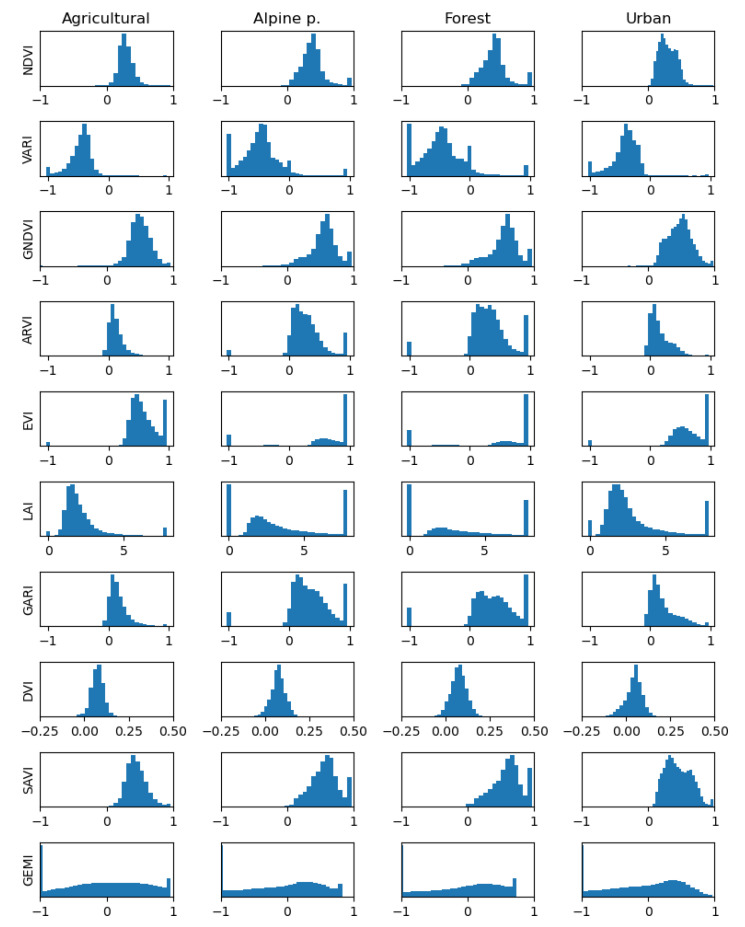
Histograms of the considered vegetation indices with respect to the considered zone. These results suggest that there is potential in using vegetation indices as discriminative features.

Figure 5 illustrates the distribution of all VI values within the dataset, which is organized into subsets based on both the index and the terrain.

#### 2.4.2. Similarity Metrics

In this work, we manually selected a number of pixels that correspond to a specific type of terrain/vegetation. All these pixels were stored in an organized structure: vectors for the first three measures and windows for the SSIM. Given the original PRISMA images, for each considered VIs, there is an image representation, out of which, based on the masks, pixels were selected. A pair of VIs results in two structures, *X* (which was always chosen to be the NDVI) and *Y* (varying from case to case).

To quantify differences between VIs, we employed four distinct similarity measures:

- **Euclidean (L2) Distance** measures the average pixel-wise differences between two index maps. Given two images *X* and *Y*, each with *n* pixels, the Euclidean distance is defined as:
  (12) $$d_{L2}\left(X,Y\right)=\sqrt{\sum_{i=1}^{n}{\left(X_{i}-Y_{i}\right)}^{2}}$$
  in the interpretation of L2 values, this is easy, as small values indicate small differences and large the contrary.
- **Pearson Correlation** captures linear dependency between two distributions. For two vectors *X* and *Y*, Pearson correlation *r* is computed as:
  (13) $$r=\frac{\sum_{i=1}^{n}\left(X_{i}-\bar{X}\right)\left(Y_{i}-\bar{Y}\right)}{\sqrt{\sum_{i=1}^{n}{\left(X_{i}-\bar{X}\right)}^{2}}\sqrt{\sum_{i=1}^{n}{\left(Y_{i}-\bar{Y}\right)}^{2}}}$$
  where $\bar{X}$ and $\bar{Y}$ are the means of *X* and *Y*, respectively.
  This Pearson correlation coefficient measures the linear dependency of the two variables: if *Y* is linearly approximated by *X*, then |r|→1; otherwise, for a weak approximation, |r|→1. If r<0, the best linear approximation is negative; when *Y* increases, *X* decreases.
- **Spearman Correlation** measures the monotonic relationship between two ranked variables. It is computed similarly to Pearson correlation, but applied to the ranks of *X* and *Y*:
  (14) $$\rho =\frac{\sum_{i=1}^{n}\left(R\left(X_{i}\right)-\bar{R(X)}\right)\left(R\left(Y_{i}\right)-\bar{R(Y)}\right)}{\sqrt{\sum_{i=1}^{n}{\left(R\left(X_{i}\right)-\bar{R(X)}\right)}^{2}}\sqrt{\sum_{i=1}^{n}{\left(R\left(Y_{i}\right)-\bar{R(Y)}\right)}^{2}}}$$
  where R(X) and R(Y) are the rank variables (i.e., storing the rank of a specific value instead of the actual value).
  The Spearman correlation coefficient quantifies the linear correlation of the rank. In other words, the two variables are Spearman-correlated if they are connected by a monotonic (including non-linear) function.
- The **Structural Similarity Index Measure (SSIM)** evaluates perceptual similarity between two images by comparing their luminance, contrast, and structural information. The SSIM between images *X* and *Y* is defined as:
  (15) $$\mathrm{SSIM}\left(X,Y\right)=\frac{\left(2\mu_{X}\mu_{Y}+C_{1}\right)\left(2\sigma_{XY}+C_{2}\right)}{\left(\mu_{X}^{2}+\mu_{Y}^{2}+C_{1}\right)\left(\sigma_{X}^{2}+\sigma_{Y}^{2}+C_{2}\right)}$$
  where
  - $\mu_{X},\mu_{Y}$ are the mean intensities;
  - $\sigma_{X}^{2},\sigma_{Y}^{2}$ are the variances;
  - $\sigma_{XY}$ is the covariance between *X* and *Y*;
  - $C_{1}$ and $C_{2}$ are small constants to stabilize the division. They are computed as $C_{1}={\left(K_{1}L\right)}^{2}$ and $C_{2}={\left(K_{2}L\right)}^{2}$, where *L* is the dynamic range of the pixel values (typically $2^{\mathrm{bits}\;\mathrm{per}\;\mathrm{pixel}}-1$), while $K_{1}=0.01$ and $K_{2}=0.03$.

The SSIM returns values between −1 and 1, where 1 indicates perfect similarity, 0 is a complete lack of similarity, and −1 indicates perfect anti-correlation. Compared to previous coefficients, SSIM is computed on windows, thus taking into account the local context and not only individual values.

The proposed approach based on multiple metrics ensures that both statistical and visual aspects of index similarity are captured, revealing hidden patterns that might be missed by a single metric.

#### 2.4.3. Statistical Discriminative Test

Given that our VI data is computed for the same location (i.e., is paired), it is possible to also run statistical tests that verify the hypothesis of two VIs being similar or not. First, we ran a normality test and since we found that the data is not normally distributed, we ran a Wilcoxon sign-ranked test to determine if VIs from a pair, for each terrain type, were similar.

**Normality test**. In this work we have used The D’Agostino–Pearson normality test [44], which is a statistical test for assessing whether a given sample does come from a normally distributed population. It combines measures of skewness and kurtosis to create a more comprehensive test of normality. The key point is that for normal (Gaussian) distribution, any moment larger than variance (including skewness and kurtosis) is zero. In this test, skewness measures asymmetry in the data distribution, while kurtosis measures the “tailedness” of the distribution; all this information is transformed to compute a chi-squared test statistic. If the resulting *p*-value is small (commonly < 0.05), it suggests that the data significantly deviates from normality.

The **Wilcoxon sign-ranked test** [45] is a non-parametric statistical test used to compare two related samples, matched samples, or repeated measurements on a single sample to assess whether their population mean ranks differ. It is often considered a non-parametric alternative to the paired *t*-test, especially when the differences between paired observations are not normally distributed. It tests whether the median difference between paired observations is zero.

### 2.5. Supervised Evaluation: Discriminative Power of VIs

#### 2.5.1. Problem Formulation

In the supervised setting, the multi-class crop recognition task is framed as semantic segmentation via pixel-wise classification. Each pixel is labeled with an agricultural crop type, and the objective is to predict these labels using machine learning classifiers trained on features derived from vegetation indices that were already discussed. All 30 crop classes were considered simultaneously. The goal was to evaluate how well vegetation indices alone could serve as discriminative features in a multi-class setting.

#### 2.5.2. Classifier and Feature Importance

This work focuses on four widely used supervised learning algorithms—Random Forest (RF), Multilayer Perceptron (MLP), Support Vector Machine (SVM), and Gradient Boosting Machine (GBM). The choice of these classifiers is motivated by the findings of Fernandez Delgado et al. [46], who, after testing over many public tabular repositories, found that Random Forest, Support Vector Machine, and Multilayer Perceptron were the best performers. Subsequent works [47,48] showed that Gradient Boosting Machine is also a very competitive tool and therefore we have included it in our set.

Although some algorithms are inherently more robust to overfitting, such as those based on ensemble learning (Random Forest and Gradient Boosting Machine) due to their ensemble averaging and randomized feature selection [49], careful hyperparameter tuning remains equally important to ensure optimal performance [50]. Since there is no universal rule for determining the best hyperparameters for a given dataset [51,52], grid search remains one of the most effective methods for identifying optimal values. This approach involves defining a discrete search space—a grid of possible values for each hyperparameter—and then training a classifier for every possible combination. Each resulting model is evaluated, and the configuration that achieves the highest performance (e.g., accuracy) is selected as the most suitable for the data at hand.

Feature Importance. In data analysis, ranking feature importance points to a task that measures the contributions of individual input variables with respect to the performance of a supervised classifier. It gains importance in explainable/interpretable AI as it assists in understanding the decision-making of a learning system and identification of key contributors in a specific problem [53]. In our scenario, we estimated the feature importance, which represents the ranking of the vegetation indices, using two methods.

First, for the tree-based classifiers (namely RF and GBM), the importance (i.e., ranking) of vegetation indices may be found as the mean and standard deviation of accumulation of the loss function (i.e., the Gini index for RF and multinomial deviance for GBM) decrease when that feature is used in a tree node [54]. Secondly, by using the permutation feature importance method [55], we can evaluate their discriminative contribution to the classification task. We recall that the permutation feature importance method assumes repeated permutations of the value of a specific feature by disabling any pre-existent relation between that feature target and measuring its impact over the learning system’s importance. Obviously, the larger the decrease in performance with respect to the baseline, the larger the importance of that feature.

Using feature importance ranking, we may identify the most discriminative vegetation indices for a specific application, such as crop identification. Furthermore, by examining the relative importance of the best and the worst, we may identify redundant (from a machine learning perspective) vegetation indices. Together, these methodological components provide a rigorous and comprehensive framework for evaluating the role, utility, and relationships among vegetation indices in both supervised and unsupervised remote sensing tasks.

### 2.6. Implementation Details

All computations were carried out in Python, version 3.10.12 using libraries such as “scikit-learn”, version 0.19.2, for classification, “numpy”, 1.21.5 and “scipy”, version 1.9.1 for index computations, and “skimage” for SSIM analysis. The PRISMA image was processed using atmospheric correction with the spectral response of the corresponding VI formulas. UAV data was preprocessed to align training and testing splits with the original dataset structure.

### 2.7. Evaluation Criteria and Comparative Scope

To ensure consistency and validity across modalities, the following evaluation criteria were adopted:

- Average Accuracy (AA): Used for supervised segmentation, representing mean class-wise accuracy.
- Index Importance Score: Derived from Random Forest feature importance values.
- Metric Consistency Across Zones: In the unsupervised setting, a focus was placed on how correlation and structural similarity varied across terrain types.
- Cross-Sensor Robustness: Insights were compared between the UAV and PRISMA platform sensors to assess whether findings hold across acquisition modalities.

## 3. Results

This section presents the experimental results obtained from both the unsupervised and supervised evaluation pipelines, reflecting the updated methodological framework. Our analysis emphasizes the statistical relationships, perceptual similarities, and classification effectiveness of a representative set of vegetation indices (VIs) computed over UAV and satellite hyperspectral datasets.

### 3.1. Unsupervised Evaluation: Inter-Index Similarity Analysis

**Figure 6:**
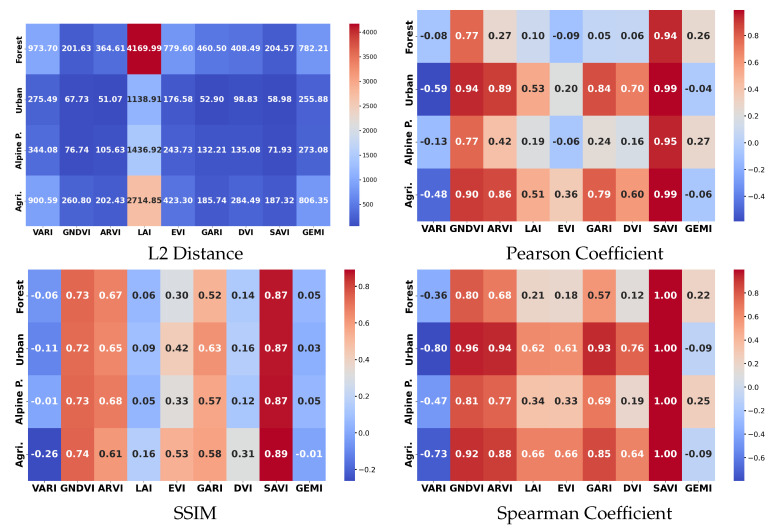
The statistical and imaging similarity of the selected vegetation indices with respect to the NDVI for various categories of landscape (agricultural land, urban, forest, pasture). The figure is better viewed in digital form, under zoom-in.

The unsupervised study focused on quantifying how closely different VIs relate to the NDVI, serving as a reference, across four semantically distinct land cover types (agricultural crop, forest, alpine pasture, and urban). As presented in the previous section, four metrics were computed: Pearson correlation, Spearman correlation, Euclidean (L2) distance, and Structural Similarity Index Measure (SSIM). The results are presented in Figure 6.

**Figure 7:**
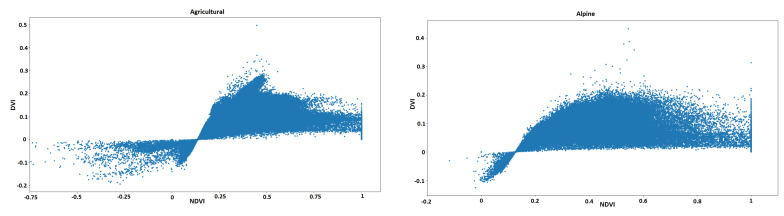

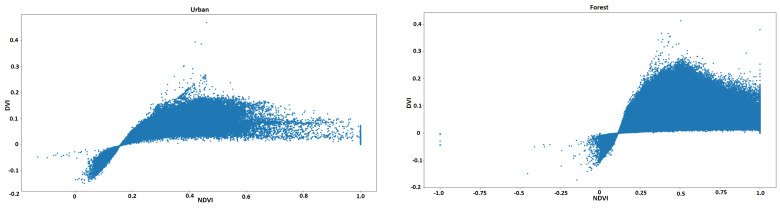
Plot of pixels in (DVI, NDVI) space for 4 types of vegetation considered. The data is more similar for “urban”, while for “forest” the points are uncorrelated.

As an overall observation and rather surprisingly, the VI values are close to each other on the “urban” landscape and more distant on the “forest” type of vegetation. However, if one examines the formulas for Vegetation Indices more closely and pays attention to their names, they will realize that they have been constructed to analyze vegetation. Therefore, the more densely covered with vegetation an area is, the more distinctive the VIs are. To enhance this observation, we also plotted points in (NDVI, DVI) space for the four zones in Figure 7: in the “urban” zone there is the most correlation, while in the “forest” zone there is the least. This result goes against previous findings [12], but we argue that our findings are more reliable since the amount of pixels used to compute statistics is much larger (approximately 15 times more, due to more images and more refined annotations).

#### 3.1.1. Distance-Based Analysis

Euclidean distances highlighted greater divergence between the NDVI and LAI across all zones, particularly in urban and alpine pasture regions. The finding suggests that the LAI, derived indirectly from the EVI, captures additional vegetation structural complexity not directly represented by the NDVI. In the opposite direction, distances to other similar VIs, such as the GNDVI and DVI, are the smallest.

With respect to landscape type, distances for urban, which contains the least vegetation from the four types, are the smallest. This is explainable by the fact that it contains small green values; thus, the overall values are smaller here, as one can notice from Figure 5, too. On the same row, one may observe that some indices show differences in the histogram shape, hinting at the possibility of discriminating between categories. In the same column, one might look for differences between indices, which would hint at their complementarity. These results suggest that there is potential in using vegetation indices as discriminative features.

However, none of the distances computed are small enough to conclude that an index represents the same information as the NDVI. For vegetation types of terrain, the indices are quite distinct, arguing for their usability as feature descriptors.

#### 3.1.2. Correlation Analysis

Across all land cover types, the NDVI exhibited the highest Pearson and Spearman correlations with the SAVI followed by the GNDVI, confirming their strong linear and monotonic dependency. In agricultural zones, Pearson coefficients between the NDVI and GNDVI exceeded 0.95, with Spearman correlations similarly high, suggesting these indices track vegetation vigor similarly under crop-dominated landscapes. The ARVI, VARI, GARI, and DVI demonstrated moderately strong correlations with the NDVI, although their performances varied more across different zones. In urban areas, correlation scores declined sharply for most indices, revealing the challenges posed by complex surface materials and mixed pixels. While no universal thresholds exist, based on our observations, we consider Pearson or Spearman coefficients above 0.9 as indicative of strong redundancy, 0.6–0.9 as partial similarity, and values below 0.6 as suggesting meaningful discriminativity between indices. These empirical ranges are consistent with interpretations in the prior literature and reflect how similarly or distinctly two indices capture vegetation variability under varying land cover types.

#### 3.1.3. Perceptual Similarity

SSIM analysis offered a complementary, perceptual perspective. While the GNDVI maintained relatively high SSIM values with the NDVI in vegetated areas (e.g., forest and agricultural fields), visual structural dissimilarities became more pronounced in urban landscapes. Indices like the VARI, designed for atmospheric resistance, maintained better SSIM in urban zones, aligning with their formulation objectives. Overall, the unsupervised findings emphasize that index relationships are highly conditioned by landscape type, with human-modified environments exposing the largest discrepancies.

To summarize our conclusions for the *unsupervised similarity* experiment, with some local exceptions, such as the relation between the NDVI and SAVI, the vegetation indices are rather dissimilar. The similarity is higher for urban landscapes, but, overall, yields rather low coefficients. These results argue for the usability of VIS features in crop recognition experiments, which are detailed in the next subsection.

#### 3.1.4. Statistical Similarity

In addition to these metrics, we have run statistical tests to determine where any indices are similar with regard to the NDVI.

First, we run the normality test. Here, the test wasn applied to each VI, over each terrain type. The largest value achieved was ≈10^−6^, which is significantly smaller that the standard threshold of p=0.05, which strongly suggests that none of the VIs, on any type of terrain, produce Gaussian distributed samples.

**Figure 8:**
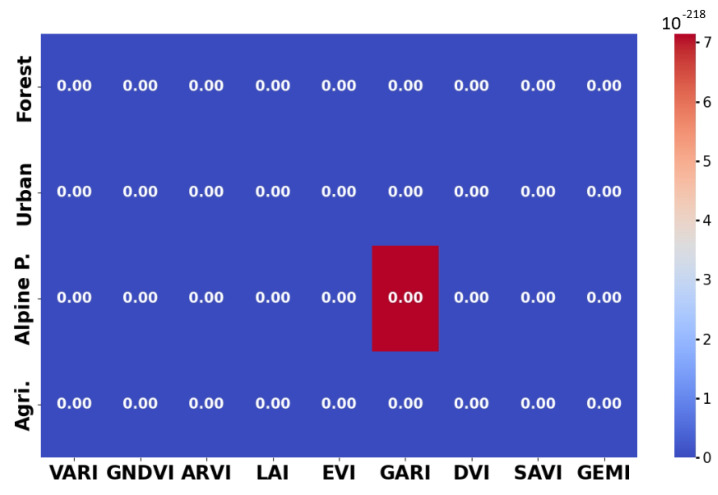
Wilcoxon values when testing for similarity between a VI and NDVI. Values smaller than 0.05 indicate disimilarity. The maximum computed value is $10^{-218}$.

Given that the data is not Gaussian and there are some dependencies, the Wilcoxon signed-rank test was also used to asses if two VIs were similar. Detailed results can be found in Figure 8. As one may notice, the obtained values are, again, far lower than the predefined threshold of p=0.05, which strongly suggests that the VIs are dissimilar.

### 3.2. Supervised Evaluation: Discriminative Power of VIs

The results were based on unsupervised examination of relations between the NDBI and each of the other vegetation indexes, and they showed that the VIs are quite distinct. We then conducted a more precise and detailed supervised analysis. In this case, we trained several classifiers, fine-tuned each of them, and analyzed the results. In the analysis, we complemented this by observing the relative feature importance and tried to determine if the relative importance was too varied, and conclude that only some indices were useful.

#### 3.2.1. Multi-Class Crop Segmentation

Using the UAV-HSI dataset, four classifiers were trained to perform semantic segmentation based on VI-derived features: RF, MLP, SVM, and GBM.

**Figure 9:**
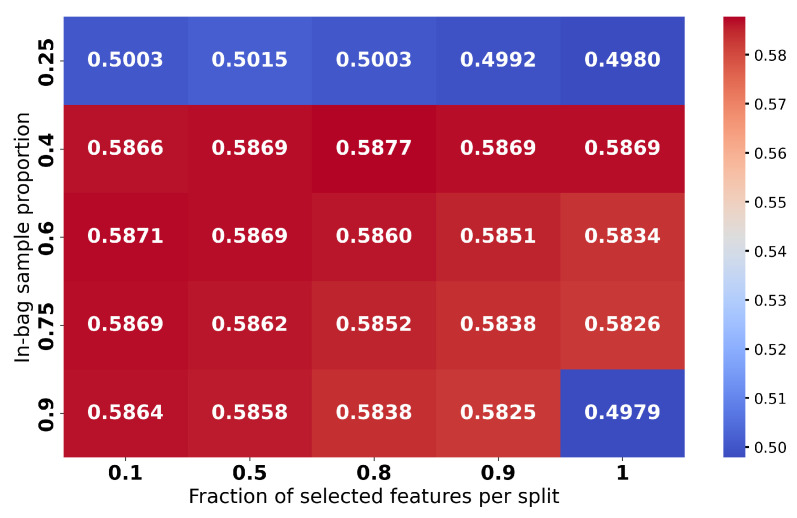
Accuracies for Random Forest under different hyperparameter (in-bag ratio and number of features considered in a node) settings.

**Figure 10:**
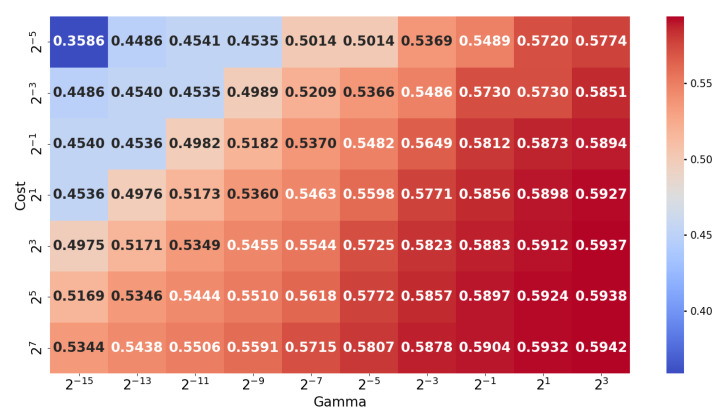
Accuracies for the Support Vector Machine under different hyperparameter (cost and γ parameter of the Gaussian kernel considered in a node) settings.

**Figure 11:**
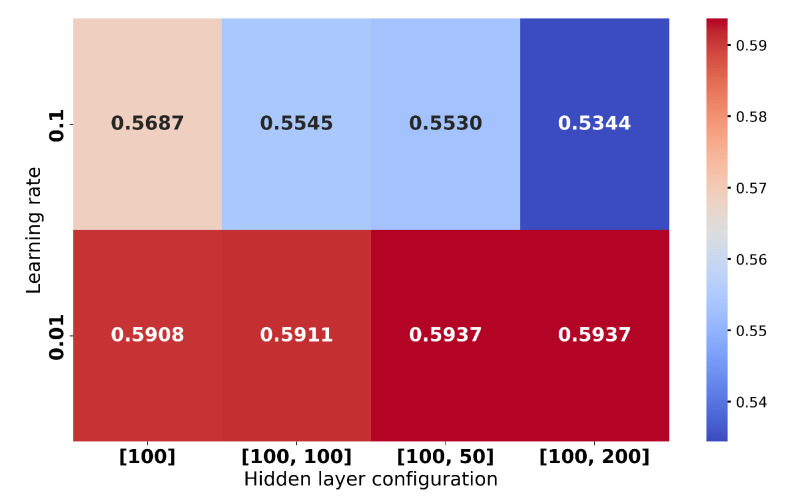
Accuracies for the Multilayer Perceptron under different hyperparameter (number of hidden layers and learning rate) settings.

**Figure 12:**
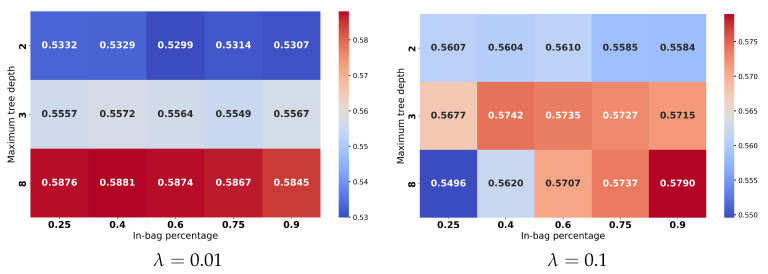
Accuracies for the different learning rates (λ) in the context of Gradient Boosting Machine.

Multiple works emphasized that each such classifier needs to go through a hyperparameter tuning process to establish the right combination for the best performance. Hyperparameters for each model were optimized via grid search to ensure fair comparisons. The detailed results, given the search in the hyperparameter space, may be followed in Figure 9, Figure 10, Figure 11 and Figure 12 and are aggregated in Table 1.

Average Accuracy (AA): Across classifiers, the average accuracy achieved using only vegetation indices ranged from 55% (SVM) to 58% (RF), relative to a baseline of 70% using the full hyperspectral cube (result reported in the previous work) [13]. None of the classifiers consistently outperformed the others, although they are rather distinct in how they determine separation. The fact that the results are similar no matter the classifier argues for the consistency of conclusions.

#### 3.2.2. Classifier Comparisons

**Figure 13:**
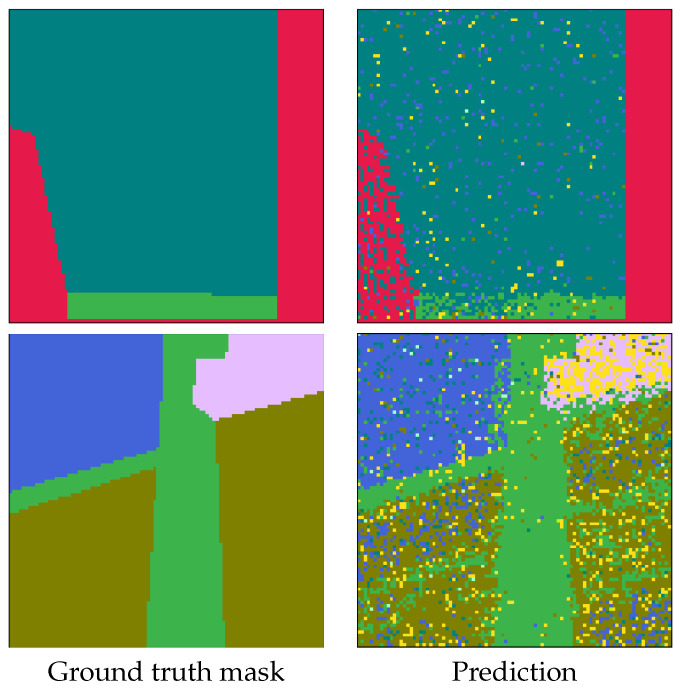
Example of pixel-wise independent prediction using Random Forest.

In summary (which is presented in Table 1), the results with considered classifiers are as follows:

- Random Forest (RF) [56]: An ensemble with 40 trees has been considered and the loss objective is the Gini index. The performance is presented in Figure 9. It reports the best average performance across all classes. Another advantage of the Random Forest is that it is highly interpretable. Using this classifier, we have also applied on a testing image to illustrate how the pixel-wise independent classification may lead to segmentation. The results may be seen in Figure 13.
- Support Vector Machine (SVM) [57]: We have an aggregated one-vs-all model with a Gaussian kernel. The achieved performance under various settings is shown in Figure 10. Overall, it underperformed relative to ensemble methods, likely due to the complex non-linear boundaries required for optimal segmentation.
- Multilayer Perceptron (MLP) [58]: We have considered a feed-forward model based on sigmoid neurons, and the performance under various settings is listed in Figure 11. Its performance, in this choice, is not the most possibly competitive. Obviously, deeper models with activation functions of the ReLU type and residual connections may improve, but while requiring larger resources.
- Gradient Boosting Machine (GBM) [59]: Its performance under different learning rates is presented in Figure 12. The results showed that it is slightly more sensitive to hyperparameter settings but is overall competitive with RF.

In addition, to obtain a better framing of the reachable performance, we continued the comparison with other works introduced in [13]. Since we consider VIs to be data descriptors, we have considered three other alternatives: RAW pixels (187 dimensional), Principal Component Analysis (PCA), and Minimum Redundancy Maximum Relevance (mRMR) [60]. Both dimensionality reduction techniques have been used to reduce the original space from the initial dimensions, 187 to 10 (which is the same with the number of VIs used). These are illustrated in the bottom part of Table 1. As one can see, the performance for VIs, although inferior, is not by much.

#### 3.2.3. Feature Importance Rankings

**Figure 14:**
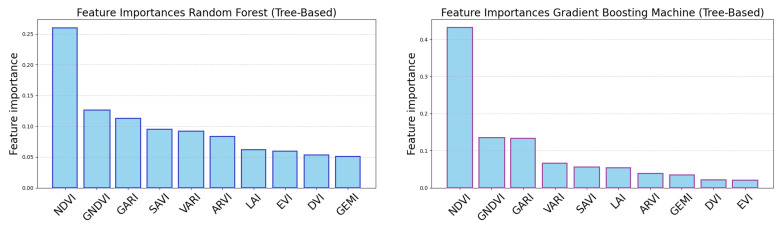
Feature importance computed as the impact of a split based on the feature in trees for Random Forest and Gradient Boosting Machine. Higher scores mean the feature is more important. While the NDVI obviously dominates, the rest are comparable.

Two methods were used to find each feature (i.e., VI)’s importance. The first method is available only for tree-based classifiers (RF and GBM) as it accumulates the impact of splits in nodes per feature. The results can be seen in Figure 14. In both cases the NDVI is found to be the most important by a noticeable margin, yet all other VIs have their impact.

The second method is computable for all classifiers. Table 2 displays the averaged permutation importance scores for each index. Permutation feature importance was applied to gauge the contribution of each index toward classification accuracy. NDVI, GNDVI, and VARI ranked consistently among the top features across classifiers and crops. Interestingly, DVI and LAI, although useful in broader vegetation assessment, ranked lower for pure crop discrimination tasks, suggesting their contribution is more nuanced.

### 3.3. Cross-Sensor Insights

The combination of UAV and PRISMA analysis reinforced several trends:

- The UAV data and the PRISMA data have been acquired in two different settings, with different sensor-to-object distances, different sensor types, etc. Consequently, the two types of data have different spatial and spectral resolutions. Yet vegetation indices behave consistently across spatial resolutions, with generalizable patterns observed between high-resolution UAV imagery and medium-spatial-resolution satellite data. These patterns include distinctiveness and comparable contributions in separation, although the NDVI remains the leader.
- Landscape type significantly mediates VI behavior. Agricultural and natural zones yield more distinctiveness among VI relationships compared to urban or mixed-use areas.
- Supervised and unsupervised results converge: indices showing stronger statistical and perceptual similarity with the NDVI also tended to perform better in classification tasks.

## 4. Conclusions

This work has investigated the usability of 10 vegetation indices as feature descriptors in discriminating among vegetation types and crops. It provided a comprehensive evaluation of VIs through both supervised and unsupervised methodologies, using two complementary hyperspectral datasets acquired from sensors mounted on UAV and PRISMA satellite platforms. By bridging statistical, perceptual, and classification-driven analyses, we have offered a multi-faceted understanding of the similarities and divergences among widely used VIs.

Our unsupervised analysis revealed that indices like the SAVI (first and foremost) and the GNDVI and ARVI (secondly) maintain strong statistical and perceptual similarities to the NDVI across most natural landscapes. Pearson and Spearman correlations confirmed close linear and monotonic relationships between the NDVI and other NIR-based indices under agricultural and forest settings. However, Euclidean distances and SSIM scores uncovered important structural and perceptual nuances, highlighting that high statistical similarity does not always imply identical visual characteristics—an important consideration in interpretation-driven applications.

The supervised experiments demonstrated that vegetation indices, despite drastically reducing feature dimensionality, can sustain strong discriminative power for multi-class and binary crop classification tasks. All classifiers performed similarly, arguing for the findings’ consistency. Notably, permutation feature importance highlighted that while the NDVI remains a dominant feature, indices like the GNDVI and VARI play critical roles, especially in specialized crop or terrain conditions.

A crucial insight from our findings is the landscape-dependent nature of VI behavior. In homogeneous, natural areas, VIs exhibit high divergences. In heterogeneous or anthropogenic settings, divergences decrease significantly, making room for redundancy. This observation emphasizes the need for adaptive index selection strategies based on land cover type, rather than relying solely on traditional, globally favored indices like the NDVI.

Another notable contribution is the demonstration that cross-sensor consistency exists: despite differences in spatial resolution and acquisition geometry between UAV and satellite platforms, the general trends in index behavior were preserved. This opens pathways for integrating UAV and satellite data into unified, multi-scale vegetation monitoring frameworks.

For discrimination purposes, several vegetation indices suit specific environmental conditions and sensor constraints. Once the NDVI is used, the SAVI is also correlated and thus redundant. In order, the GEMI, VARI, and DVI are the most distinct fromthe NDVI, and for a general problem, they are the best suggestion. For discrimination, VIs are classifier- and crop-dependent. But as a rule of thumb, in order, the LAI (which by definition is distinct), GNDVI, and VARI contribute to most of the discrimination. On the opposite side, the DVI and GEMI have the weakest contribution, and thus are the least useful.

However, this study is not without limitations. Our evaluation focused predominantly on indices derived from VIs and NIR bands, with limited exploration of SWIR or thermal-sensitive descriptors. Moreover, seasonal variations, different crop growth stages, and broader geographic diversity warrant future investigation to generalize findings across global agricultural systems. Our primary focus was on assessing the statistical distinctiveness and discriminative power of VIs using data acquired at specific time points. As such, seasonal variation remains outside the scope of this work and represents a potential direction for future research. Expanding the scope to include radar- and LiDAR-based vegetation metrics also represents a promising direction for future research.

Another limiting point is that in this study we have adopted a data analysis perspective first, in which physical and biological consideration were overlooked; the VIs have been treated more as black boxes that generate data than carefully analyzing physical relations. The motivation is two-fold. Firstly, data analysis has reached a level of maturity where independent conclusions can be discovered. Secondly, prior work is more detailed in terms of physical and biological perspectives but rather lacking in a data-science approach.

Considering these insights, several recommendations emerge for practitioners and researchers:

- Multi-index approaches should be preferred over reliance on a single VI, especially for heterogeneous or complex landscapes. Similar feature importance is low and values for similarity argue for VI diversity.
- Supervised classification workflows show convergent results, although sensors of a different nature have been used.
- Perceptual metrics like SSIM provide valuable complementary information alongside statistical correlations in index evaluation.
- Future VI development should consider both biophysical sensitivity and perceptual distinctiveness, tailoring indices to application-specific demands.

In conclusion, vegetation indices, though simple in formulation, encapsulate complex interactions with vegetation biochemistry, structure, and environment. A nuanced understanding of their behavior, supported by rigorous evaluation across sensors and landscapes, is essential for building the next generation of remote sensing applications aimed at addressing pressing challenges in agriculture, forestry, and environmental stewardship.

## Figures and Tables

**Table 1:** Comparison of performances (accuracy measured as [%]) between the best setting for each classifier. The comparison included various features.

Classifier	RF	SVM	MLP	GBM
**VIs**	58.71	59.42	59.37	58.81
**RAW**	69.98	60.84	63.25	61.17
**PCA**	39.89	63.23	69.35	64.74
**mRMR**	56.47	61.44	61.79	43.31

**Table 2:** Permutation-based measuring of the feature importance (of vegetation indices) with each of the four classifiers considered. We report the importance mean (“Mean”—higher values mark more importance) and importance standard deviation (“Std”—where smaller values mean that the computed importance is more stable with regard to consecutive runs).

Feature	Mean	Std	Feature	Mean	Std
NDVI	0.3027	0.00054	LAI	0.4959	0.0012
GNDVI	0.1077	0.00043	EVI	0.4940	0.0011
GARI	0.0895	0.00032	SAVI	0.4147	0.0018
VARI	0.0864	0.00039	NDVI	0.4033	0.0014
SAVI	0.0503	0.00032	GEMI	0.3858	0.0017
ARVI	0.0499	0.00027	DVI	0.3596	0.0019
GEMI	0.0388	0.00024	GARI	0.2598	0.0019
EVI	0.0219	0.00021	GNDVI	0.2472	0.0019
LAI	0.0214	0.00027	VARI	0.1950	0.0016
DVI	0.0202	0.00024	ARVI	0.1705	0.0013
**Random Forest**			**Gradient Boosting Machine**		
**Feature**	**Mean**	**Std**	**Feature**	**Mean**	**Std**
NDVI	0.3299	0.00059	LAI	0.3169	0.00298
GNDVI	0.2984	0.00046	NDVI	0.2935	0.00202
VARI	0.2637	0.00051	ARVI	0.27355	0.00161
SAVI	0.2559	0.00055	VARI	0.2663	0.00120
DVI	0.2018	0.00058	GNDVI	0.2307	0.00225
LAI	0.2004	0.00038	EVI	0.2303	0.00173
EVI	0.1674	0.00042	SAVI	0.2099	0.00094
GARI	0.1285	0.00043	GARI	0.2093	0.00178
ARVI	0.0874	0.00038	GEMI	0.1314	0.00120
GEMI	0.0448	0.00020	DVI	0.1190	0.00189
**Multilayer Perceptron**			**Support Vector Machine**		

## Data Availability

The datasets generated and/or analyzed during the current study are available from the corresponding authors on reasonable request. The UAV-HSI dataset is publicly available at https://www.scidb.cn/en/detail?dataSetId=6de15e4ec9b74dacab12e29cb557f041 (accessed on 16 June 2025) and the PRISMA data can be accessed through the ASI-PRISMA portal (https://prisma.asi.it/) (accessed on 16 June 2025). Where restrictions apply to data sharing (e.g., proprietary annotations), data will be made available by the authors upon reasonable request.

## Abbreviations

The following abbreviations are used in this manuscript:

AA	Average Accuracy
ARVI	Atmospherically Resistant Vegetation Index
DVI	Difference Vegetation Index
EVI	Enhanced Vegetation Index
GBM	Gradient Boosting Machine
GEMI	Global Environmental Monitoring Index
GNDVI	Green Normalized Difference Vegetation Index
LAI	Leaf Area Index
MLP	MultiLayer Perceptron
mRMR	Minimum Redundancy Maximum Relevance
NDVI	Normalized Difference Vegetation Index
NIR	Near Infrared
PCA	Principal Component Analysis
RF	Random Forest
RS	Remote Sensing
SAVI	Soil-Adjusted Vegetation Index
SSIM	Structural Similarity Index
SVM	Support Vector Machine
UAV	Unmanned Aerial Vehicle
VARI	Visible Atmospherically Resistant Index
VI	Vegetation Index
